# Rapid Visual Detection of Mycoplasma Hominis Using an RPA-CRISPR/Cas12a Assay

**DOI:** 10.3390/bios15120821

**Published:** 2025-12-18

**Authors:** Jie Chen, Shutao Liu, Sunyi Chen, Jingwen Mai, Maiwula Abudukadi, Yao Chen, Jie Lu, Guanglei Li, Chenchen Ge

**Affiliations:** 1 College of Health Science and Environmental Engineering, Shenzhen Technology University, 3002 Lantian Road, Pingshan District, Shenzhen 518118, China; 202200501096@stumail.sztu.edu.cn; 2 Department of Radiology and Nuclear Medicine, Xuanwu Hospital, Capital Medical University, Beijing 100053, China; lst41@sina.com (S.L.); imaginglu@hotmail.com (J.L.); 3 Department of Dermatology, Huashan Hospital, Fudan University, Shanghai 200040, China; csyysc28@163.com; 4 KingMed School of Laboratory Medicine, Guangzhou Medical University, Guangzhou 511436, China; 2023112115@stu.gzhmu.edu.cn (J.M.); 2021141087@stu.gzhmu.edu.cn (M.A.); 2023112113@stu.gzhmu.edu.cn (Y.C.); 5 College of Pharmacy, Shenzhen Technology University, 3002 Lantian Road, Pingshan District, Shenzhen 518118, China

**Keywords:** RPA, CRISPR/Cas12a, lateral flow assay, visual detection

## Abstract

Mycoplasma hominis (MH) is a prevalent opportunistic pathogen that is strongly associated with a wide range of urogenital tract infections and severe adverse pregnancy outcomes in clinical settings. Current MH detection methods, including microbial culture and qPCR, are time-consuming and rely on complex equipment, making them unsuitable for scenarios requiring rapid or simplified testing. In this study, we developed a visual readout biosensing platform by synergistically integrating recombinase polymerase amplification (RPA), CRISPR/Cas12a-mediated target nucleic acid recognition, and lateral flow biosensors for the rapid, sensitive, and specific identification of MH. The assay specifically targets the MH-specific 16S rRNA gene, achieving a limit of detection as low as 2 copies/reaction of recombinant plasmid containing the target gene with a total assay time of 60 min. Critical reaction parameters, including Cas12a-crRNA molar ratio, volume of RPA amplicon input, and Cas12a cleavage time, were systematically optimized to maximize the biosensor’s response efficiency and detection reliability. The platform exhibited exceptional specificity, with no cross-reactivity observed against common co-occurring urogenital pathogens, and effectively minimized aerosol contamination risks via a rigorous decontamination workflow. Furthermore, this work represents the first documented implementation of a contamination-control protocol for an MH-specific CRISPR-LFA assay. Notably, testing results from 18 clinical samples demonstrated the high specificity of this assay, highlighting its promising potential for clinical application.

## 1. Introduction

Mycoplasma hominis (MH), a cell wall-deficient microorganism, is primarily transmitted via sexual contact or vertical transmission in clinical settings [1]. As an opportunistic pathogen, it exerts distinct clinical significance in specific susceptible populations and pathological contexts [2]. When co-infecting with other microorganisms such as *Gardnerella vaginalis*, MH can contribute to the development of bacterial vaginosis or preterm birth and other adverse perinatal events [3,4]. Furthermore, post-partum or post-abortal endometritis has also been frequently attributed to MH infection [5]. In immunocompromised individuals, such as kidney transplant recipients or low-birth-weight neonates, MH induces distinct urinary tract infections and extragenital infections, respectively [6,7,8]. These clinical manifestations collectively underscore the imperative of timely MH detection and targeted therapeutic interventions to curb pathogen transmission and minimize associated adverse clinical outcomes.

Currently, routine clinical detection of MH primarily relies on traditional culture-based methods and quantitative real-time PCR (qPCR). Although microbial culture is recognized as the diagnostic gold standard for MH, it is plagued by prolonged incubation cycles and a high propensity for false-negative results, hindering timely clinical decision-making. Nonetheless, its diagnostic performance can be substantially enhanced when coupled with mass spectrometry [9] or 16S rDNA sequencing [10]. As the most widely adopted molecular diagnostic tool for nucleic acid detection, qPCR enables the identification of nearly all known pathogenic microorganisms and has been validated for the diagnosis of urogenital MH infections in clinical practice [11,12,13]. Nonetheless, qPCR requires sophisticated thermocycling instrumentation and operation in accredited molecular diagnostic laboratories, which severely limits its accessibility for scenarios requiring rapid or simplified testing. Considering these limitations, there is an urgent unmet clinical need to develop a rapid and user-friendly detection platform to improve MH diagnostic capacity across heterogeneous clinical settings.

Recombinase Polymerase Amplification (RPA) is a promising isothermal nucleic acid amplification technique enables rapid, on-site target nucleic acid detection. Compared to conventional PCR, RPA offers two principal advantages: (1) it eliminates the requirement for complex thermal cycling apparatus, thereby rendering it particularly suitable for simplified testing protocols; (2) it achieves faster detection by circumventing the necessity for repeated thermal cycling, cutting down the overall assay time by 50% or more in most cases. However, the isothermal amplification process at relatively low constant temperatures may increase the risk of non-specific amplification products [14,15]. To address this critical limitation and improve detection fidelity, a specific amplification product detection module can be integrated downstream of the RPA reaction. The CRISPR/Cas12a system has emerged as a potent tool to enhance both the specificity and sensitivity of isothermal amplification product detection in biosensing platforms. This system leverages the synergistic activity of the Cas12a enzyme and CRISPR-related RNA (crRNA) to mediate highly precise, sequence-specific nucleic acid cleavage. The CRISPR/Cas12a platform has been successfully employed for the detection of a wide range of targets, including viral pathogens, bacterial pathogens, and small molecules [16,17,18,19].

Based on the previously discussed research, this manuscript introduces a visual biosensing platform for MH that integrates RPA, CRISPR/Cas12a, and lateral flow biosensors to achieve rapid and user-friendly target identification. To address the critical issue of aerosol contamination from RPA amplicons-a common challenge in open-cap detection-this study presents the first MH-specific CRISPR-LFA method that incorporates a documented contamination-control protocol. Furthermore, we have developed a safe and efficient decontamination method to address aerosol contamination derived from RPA amplicons [20,21]. This safe and efficient decontamination method successfully eliminates contamination, thereby ensuring experimental reliability even in laboratory settings lacking dedicated physical partition facilities.

## 2. Materials and Methods

### 2.1. Reagents and Materials

The RPA kit was purchased from Jiangsu Qitian Gene Biotechnology Co., Ltd. (Wuxi, Jiangsu, China). Sodium phosphate tribasic anhydrous (Na_3_PO_4_), sucrose, agarose, boric acid (H_3_BO_3_), borax decahydrate (Na_2_B_4_O_7_·5H_2_O), and Triton X-100 were purchased from Macklin Biochemical Technology Co., Ltd. (Shanghai, China). Tween 20, sodium chloride (NaCl), and trisodium citrate (C_6_H_5_Na_3_O_7_) were purchased from Shanghai Aladdin Biochemical Technology Co., Ltd. (Shanghai, China). 6 × DNA loading buffer and 0.1–10 kb DNA ladder were purchased from Beyotime Biotechnology Co., Ltd. (Shanghai, China). Polyethylene glycol 4000 (PEG 4000) and bovine serum albumin (BSA) were purchased from Solarbio Science & Technology Co., Ltd. (Beijing, China), and chloroauric acid (HAuCl_4_) was purchased from Shenzhen Kejie Industrial Development Co., Ltd. (Shenzhen, China). IgG fraction monoclonal mouse anti-digoxin was purchased from Jackson ImmunoResearch (West Grove, PA, USA). Streptavidin (SA) was supplied by Bioss Antibodies Inc. (Beijing, China). Polyclonal goat anti-mouse IgG (H + L) was purchased from Baiaotong Experimental materials center (Luoyang, China). NEBuffer r2.1 and Cas12a protein were purchased from New England Biolabs (Massachusetts, USA). Reporter probe, crRNA, zero background TOPO-TA cloning kit, blunt/TA DNA A-Tailing kit, and phosphate-buffered saline (PBS) were purchased from Sangon Biotech Co., Ltd. (Shanghai, China). The agarose gel extraction kit was acquired from Omega Bio-tek (Norcross, GA, USA), and the plasmid miniprep kit (spin-column) was obtained from Tiangen Biotech (Beijing, China). The magnetic bead-based nucleic acid extraction kit was sourced from Tiangen Biotech (Beijing, China). Liquid quality control materials for MH (high concentration), *Chlamydia trachomatis* (CT, high concentration), *Neisseria gonorrhoeae* (NG, high concentration), and *Mycoplasma genitalium* (MG, high concentration) were purchased from Zhongke Quality Inspection (Beijing, China). Nucleic acid decontamination reagent was purchased from Tsingke Biotech Co., Ltd. (Beijing, China).

### 2.2. Preparation of Gold Nanoparticles (AuNPs) Colloidal Solution

Monodisperse gold nanoparticles with an average diameter of 13 nm were synthesized using the sodium citrate reduction method [22]. Briefly, 100 mL of a 0.01% (*w*/*v*) HAuCl_4_ solution was heated to boiling under vigorous magnetic stirring (180 °C, 1600 rpm). Subsequently, 4 mL of a 1% sodium citrate solution was rapidly injected into the boiling suspension. The reaction was allowed to proceed until a color change to wine red, indicating nanoparticle formation. The solution was maintained at boiling for an additional 10 min to ensure complete reduction in chloroauric acid and robust stabilization of the AuNP colloids. After removing the heat source, the mixture was continuously stirred until it cooled to room temperature naturally. The resulting colloidal gold suspension was then stored at 4 °C under light-shielded conditions.

### 2.3. Preparation of AuNPs-Antibody Conjugates

Six microliters of 0.1 M K_2_CO_3_ were added to 1 mL of AuNPs solution and mixed 5 min to adjust the colloidal pH for optimal antibody immobilization. Next, 10 µg of anti-digoxin antibody was added and mixed at 100 rpm for 30 min at room temperature. To block nonspecific binding sites on the AuNP surface, 100 µL of 10% BSA was added, followed by mixing at 100 rpm for 30 min. The AuNP-antibody solution was centrifuged at 13,400× *g* for 20 min and washed three times with a rinsing buffer (20 mM Na_3_PO_4_, 5% BSA, 0.25% Tween-20, and 10% sucrose) to remove unbound antibodies. The AuNP-antibody pellets were resuspended in 100 µL of rinsing buffer and stored at 4 °C.

### 2.4. Preparation of Lateral Flow Strips

Sample pads were prepared by soaking glass fiber membranes in borate buffer (containing 4% Triton X-100, 1.8% NaCl, 1% BSA, and 2% PEG 4000, pH 9.0) for 30 min. AuNP-digoxin antibody conjugates were sprayed onto the glass fiber at 15 µL/cm to create conjugate pads. Streptavidin (1 µg/µL) and goat anti-mouse IgG (1 µg/µL) were sprayed onto a nitrocellulose (NC) membrane to form test zones (T zones) and control zones (C zones), respectively. The T zones and C zones were spaced 5–8 mm apart. The NC membrane was dried at 37 °C and stored under desiccated conditions. All components were dried at 37 °C and stored in environment with relative humidity below 25%. The sample pad, conjugate pad, NC membrane, and absorbent pad were aligned on an adhesive plate with 2–3 mm overlaps to ensure unimpeded capillary-driven sample flow, cut into 4 mm wide strips with a strip cutter (Hangzhou Fenghang Technology Co., Ltd., Zhejiang, China) and stored in a dry, sealed container for subsequent biosensing assays.

### 2.5. Evaluation of Amplification Efficiency of RPA Primer Sets by the RPA-CRISPR/Cas12a Detection System

The sequences of three RPA primer sets targeting the MH 16S rRNA gene are listed in Table S1. Three pairs of RPA primers were used to amplify the target DNA fragment, respectively. Each 45 µL amplification reaction consisted of 25 µL Buffer V, 2 µL each of 10 µM forward and reverse primers, 2 µL of the MH genomics liquid quality control, 5 µL of magnesium acetate (280 mM), and nuclease-free water to the final volume. The reaction mixture was then incubated at 37 °C in a water bath for 20 min to generate amplicons. Their amplification performance was initially tested with agarose gel electrophoresis and further validated using a CRISPR/Cas12a cleavage-based sensing assay.

For the RPA-CRISPR/Cas12a fluorescent detection system, the reaction mixture contained 4 µL of RPA amplicons, 0.5 µL of 20 µM crRNA, 0.8 µL of 10 µM Cas12a, 0.8 µL of 20 µM FQ reporter probe, 2 µL of Buffer 2.1, and nuclease-free water to the final volume. Fluorescence signals (FAM channel) were recorded every 30 s for 120 cycles at 37 °C using a real-time PCR instrument (Thermo Fisher Scientific, Waltham, MA, USA). Data were analyzed with GraphPad Prism 8.0.2.263. For the RPA-CRISPR/Cas12a visible detection system, each 20 µL reaction consisted of 6 µL of RPA amplicons, 0.5 µL of 20 µM crRNA, 0.8 µL of 10 µM Cas12a, 0.8 µL of 20 µM bio-dig reporter probe, 2 µL of Buffer 2.1, and nuclease-free water to the final volume. After incubation at 37 °C for 30 min, 60 µL of PBS was added to the RPA-CRISPR/Cas12a reaction mixture, resulting in a total volume of 80 µL. The mixture was then loaded onto the sample pad of the lateral flow strip. After 15 min, the test line was visually examined, and its grayscale intensity was recorded using a colloidal gold immunochromatographic reader. The sequences of the crRNA, FQ reporter probe, and bio-dig reporter probe are listed in Table S1.

### 2.6. Construction of Recombinant Plasmid Standard

Nucleic acid fragments of the MH 16S rRNA gene were PCR-amplified using specific primers, separated via 2% agarose gel electrophoresis, and purified with a PCR purification kit. The concentration and purity of the PCR amplicon were quantified using a NanoDrop spectrophotometer (Thermo Fisher Scientific, Waltham, MA, USA). The fragments (154 bp) were then inserted into the pUCI-T vector (1865 bp) through TA cloning and transformed into *E. coli* DH-5α competent cells. Positive clones were selected on LB agar plates supplemented with ampicillin, sequenced, and those with the correct insert were cultured for plasmid amplification. The recombinant plasmids were extracted, purified, and their concentrations and purity were verified with the NanoDrop before storage at −20 °C.

### 2.7. Specificity Evaluation of the MH Biosensing Assay

Genomic DNA from CT, NG, MG, and MH from certified liquid quality control materials was extracted using a commercial magnetic bead-based nucleic acid kit. The genomes were quantified, and equal concentrations were added to the RPA reaction system, followed by incubation at 37 °C for 20 min to enable target amplification. The RPA amplicons were then transferred to the CRISPR/Cas12a detection system and incubated at 37 °C for 30 min. The mixture was applied to a lateral flow strip, where color development at the T zone was observed within 10–15 min. The T zone’s gray value was semi-quantitatively measured using an immunochromatographic strip reader. Data were analyzed with GraphPad Prism 8.0.2.263.

### 2.8. Sensitivity Evaluation of the MH Biosensing Assay

For sensitivity analysis, a 10-fold serial dilution series of the recombinant plasmid standard were prepared, ranging from 10^5^ to 1 copies/µL. Two microliters of each dilution were added to the RPA reaction, incubated at 37 °C for 20 min, then mixed with the CRISPR/Cas12a system and incubated for another 30 min. The reaction mixture was diluted with PBS, applied to a lateral flow strip, and developed for 10–15 min at room temperature. The T zone’s color intensity was visually assessed and semi-quantitatively measured using an immunochromatographic strip reader. Data were analyzed with GraphPad Prism 8.0.2.263.

### 2.9. Clinical Sample Validation of the Biosensing Platform

Four healthy (H) specimens, one *Ureaplasma urealyticum* (UU) and MG coinfected specimen, one UU and CT coinfected specimen, one UU-monoinfected clinical specimen, one CT-monoinfected clinical specimen, three MH-monoinfected clinical specimens, six MH and UU coinfected specimen, and one MH and MG coinfected specimen were collected from Huashan Hospital, Shanghai (Ethics Approval No. 2023-651). Genomic DNA from clinical samples was extracted and tested using the integrated RPA/CRISPR-Cas12a-lateral flow visual biosensing platform. The signal intensity of the T zone was quantitatively detected using an immunochromatographic strip reader and data were statistically analyzed with GraphPad Prism 8.0.2.263.

## 3. Results

### 3.1. Working Principle of the MH Visual Biosensing Assay

**Figure 1:**
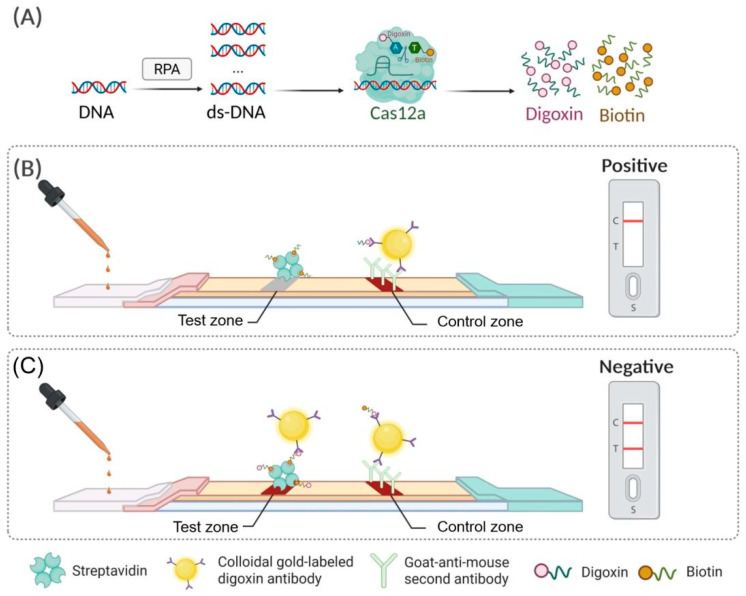
Schematic diagram of the visual MH biosensing platform based on RPA-CRISPR/Cas12a. (**A**) RPA-mediated isothermal amplification of the MH-specific 16S rDNA target sequence. (**B**) Lateral flow biosensor readout for MH-positive samples and (**C**) MH-negative samples.

The working principle of the visual biosensing assay for MH is schematically is depicted in Figure 1. This assay utilizes RPA-mediated isothermal amplification of a MH-conserved 16S rRNA gene fragment, which is subsequently combined with a preassembled CRISPR/Cas12a nucleic acid recognition system (Figure 1A).

Upon the presence of MH-specific target DNA, the RPA amplicon is identified by a sequence-specific crRNA, triggering the activation of Cas12a’s dual nuclease activity. This activation leads to the cis-cleavage of the target amplicon and the collateral trans-cleavage of biotin-digoxin (bio-dig) reporter probes. The resulting cleavage products are then applied to an AuNP-based lateral flow strip for visual signal readout. Complete cleavage of the bio-dig probes allows the digoxin moiety to bind to anti-digoxin antibody-conjugated AuNPs, forming AuNP-anti-digoxin antibody-digoxin complexes. These complexes migrate along the strip through capillary action and are captured by a goat anti-mouse IgG at the control zone (C zone), producing a visible red band via AuNP aggregation. Under these conditions, the test zone (T zone) remains colorless due to the lack of intact reporter probes available for biotin-SA interaction-mediated capture (Figure 1B). In the absence of the MH target DNA, Cas12a remains catalytically inactive, and the bio-dig reporter probe remains intact. The intact probe binds to the anti-digoxin antibody on the AuNPs, forming an AuNP-anti-digoxin antibody-bio-dig reporter probe complex. This complex migrates along the strip and is captured at the T zone through interaction with pre-immobilized SA, producing a visible red band via AuNP accumulation. Excess AuNP-antibody conjugates continue to migrate to the C Zone, where they are captured by the secondary antibody, forming a second red band (Figure 1C).

In summary, the detection is based on Cas12a trans-cleavage activity, which leads to a reduction in test line signal as the target MH concentration increases. For qualitative analysis, results can be directly interpreted by visually observing the red color intensity of the T zone. For semi-quantitative analysis, the grayscale value of the T zone can be measured using a lateral flow strip reader.

### 3.2. Optimization of the Detection Conditions for the Lateral Flow Strip

Initially, monodisperse AuNPs were synthesized via the trisodium citrate reduction method. The synthesized AuNPs were comprehensively characterized by UV-visible spectrophotometry, dynamic light scattering (DLS), and transmission electron microscopy (TEM) to verify their morphology, size uniformity, and colloidal stability. As depicted in Figure S1a, the gold nanoparticles exhibited a maximum absorption peak at approximately 520 nm (absorbance value of 0.8141) and displayed a typical wine-red color, indicative of well-dispersed colloidal AuNPs with no significant aggregation. DLS analysis (Figure S1b) indicated a narrow size distribution centered at ~13 nm, and this result was further confirmed by TEM imaging, which revealed spherical AuNPs with homogeneous particle dimensions (Figure S1c,d). Collectively, these characterization results confirm the successful synthesis of monodisperse 13 nm AuNPs suitable for application in the lateral flow biosensor.

**Figure 2:**
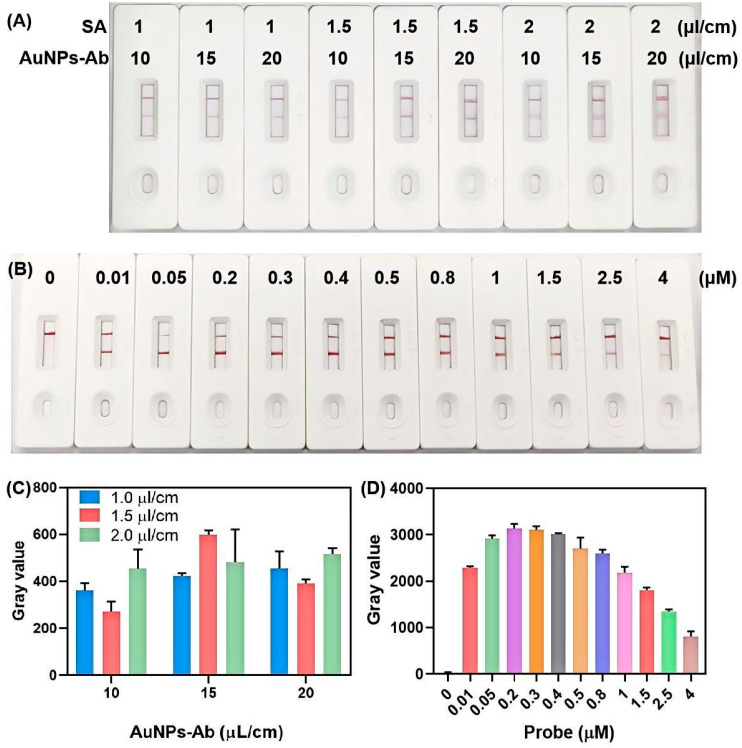
Optimization of lateral flow strip detection conditions. (**A**) Visual results and (**C**) corresponding quantitative grayscale analysis from orthogonal experiments with varying SA spray concentrations and AuNP-antibody conjugate loading concentrations. (**B**) Visual results and (**D**) quantitative grayscale analysis with different concentrations of bio-dig probe. Error bars indicate the standard deviation from three independent measurements.

For the lateral flow strip detection system, experimental condition optimization was performed to minimize false-positive and false-negative signals while ensuring sufficient color intensity at the T zone for visual interpretation of biosensing results. The key parameters selected for optimization include: (1) the concentration of SA immobilized on the T zone, (2) the loading concentration of AuNP-antibody conjugates on the conjugate pad, and (3) the working concentration of the bio-dig reporter probe in the CRISPR/Cas12a reaction system. Orthogonal experimental results (Figure 2A) indicated that the most pronounced color development and highest grayscale value at the T zone were achieved with an SA spray concentration of 1.5 µL/cm and an AuNPs-antibody conjugate loading concentration of 15 µL/cm (Figure 2C). These conditions were therefore selected as the optimal parameters for all subsequent biosensing experiments.

Figure 2B illustrates the variation in T zone color intensity in response to different concentrations of the bio-dig probe. As the probe concentration increased from 0 to 0.2 µM, both the color intensity and grayscale value of the T zone increased in a concentration-dependent manner (Figure 2D). However, further increasing the probe concentration from 0.2 µM to 4 µM resulted in a gradual decrease in signal intensity due to the classic hook effect. This phenomenon occurred because excess probe competitively occupied the binding sites on both the digoxin antibody and the immobilized SA, thereby inhibiting the formation of AuNP-Ab/bio-dig probe/SA sandwich immunocomplex at the T zone. Based on these findings, the optimal concentration of the bio-dig probe was determined to be 0.2 µM.

### 3.3. Evaluation of RPA Primer Amplification Efficiency and Corresponding CRISPR/Cas12a Sensing Compatibility

**Figure 3:**
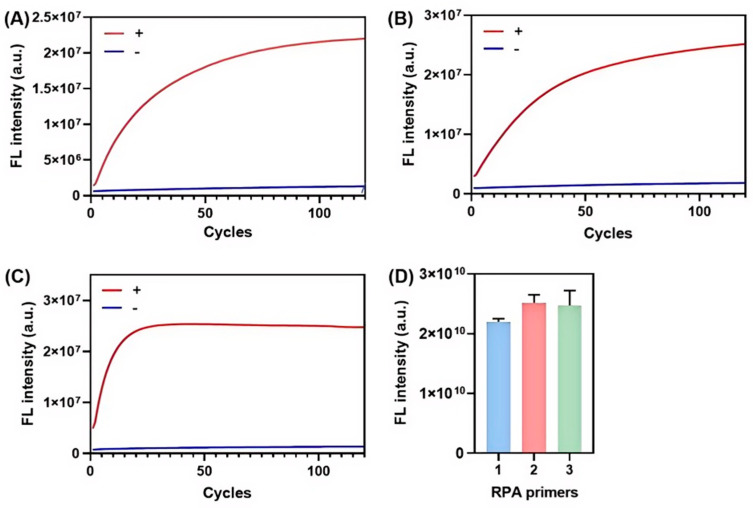
Evaluation of RPA primer amplification efficiency via Cas12a trans-cleavage triggered by RPA amplicons. (**A**–**C**) Real-time fluorescence kinetic curves of MH detection using the RPA-CRISPR/Cas12a fluorescent sensing system with primer set 1/crRNA1, primer set 2/crRNA2, and primer set 3/crRNA3, respectively. (**D**) End-point fluorescence intensity quantification at 120 cycles (30 sec each) for each primer-crRNA combination, with error bars representing standard deviations from three independent tests.

Three crRNA sequences were designed based on the target regions of the amplification products generated by three distinct RPA primer sets targeting the MH 16S rRNA gene (sequences listed in Table S1). To comprehensively evaluate the amplification efficiency of these RPA primers and their subsequent compatibility with the CRISPR/Cas12a sensing module, we performed 2% agarose gel electrophoresis and assessed the Cas12a trans-cleavage activity triggered by the RPA amplicons. Electrophoretic analysis confirmed that all three primer sets successfully yielded target-specific bands of the anticipated molecular weight (Figure S2). The results of real-time fluorescence detection using the CRISPR/Cas12a system are shown in Figure 3.

As demonstrated in Figure 3, all RPA amplification products activated the trans-cleavage activity of Cas12a, leading to the release of detectable fluorescent signals via the cleavage of the FQ probe. Specifically, the reactions utilizing primer set 1 with crRNA1 and primer set 2 with crRNA2 reached a plateau at approximately 100 cycles (Figure 3A,B). In contrast, the combination of primer set 3 and crRNA3 reached the signal plateau within 30 cycles (Figure 3C), demonstrating a significantly faster signal rise rate than the other two primer-crRNA pairs.

Kinetic analysis provided additional validation for these observations. The Michaelis-Menten constants (Km) were calculated as 26.17 for primer set 1, 27.54 for primer set 2, and 3.826 for primer set 3. A lower Km value indicates a higher binding affinity between the Cas12a-crRNA complex and the target amplicon. These findings demonstrate that primer set 3 exhibited the highest amplification efficiency, not only confirm that primer set 3 produced the largest quantity of target amplicons within the same timeframe but also that its products had stronger affinity for the CRISPR/Cas12a recognition system. As a result, the increased quantity of RPA amplification products facilitated a more rapid activation of Cas12a trans-cleavage activity. Moreover, analysis of the fluorescence signal plateaus revealed that both primer set 2/crRNA2 and primer set 3/crRNA3 generated stronger signals. Based on its superior amplification performance and efficiency, primer set 3 was selected for all subsequent MH biosensing experiments.

### 3.4. Optimizing of RPA-CRISPR/Cas12a Biosensing Assay Detection Conditions

**Figure 4:**
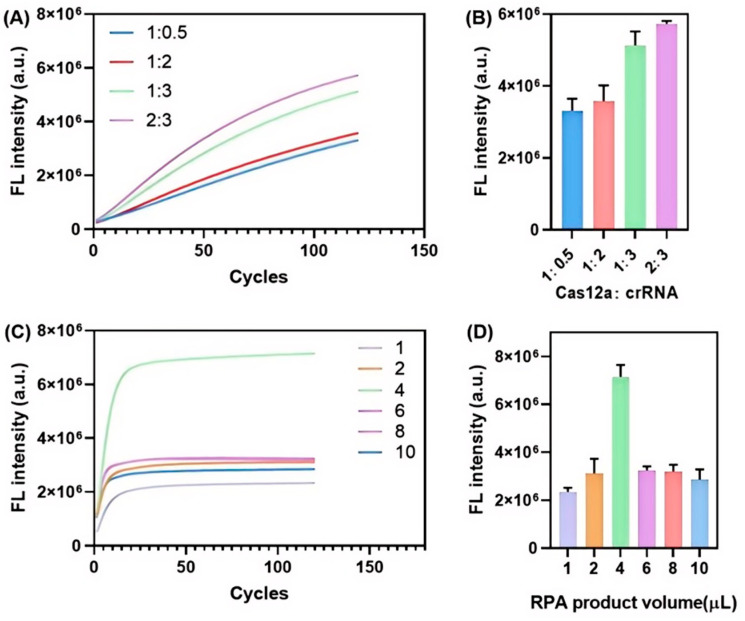
Optimization of Cas12a-to-crRNA molar ratio and the impact of RPA amplicon volume on Cas12a collateral cleavage activity. (**A**) Time-dependent fluorescence kinetic profiles for various Cas12a-crRNA molar ratios; (**B**) End-point fluorescence intensity at cycle 120 for each ratio; (**C**) Real-time fluorescence kinetic curves with different RPA amplicon volumes; (**D**) End-point fluorescence intensity at cycle 120 for each amplicon volume. Error bars represent the standard deviation from three independent tests.

The collateral trans-cleavage efficiency of Cas12a directly determines the cleavage degree of the bio-dig reporter probe, subsequently impacting the colorimetric signal intensity at the T zone of the lateral flow biosensor. To improve the overall biosensing performance, we conducted a systematic optimization of three key parameters: (1) the molar ratio of Cas12a to crRNA, (2) the volume of RPA amplicon added into the CRISPR/Cas12a reaction system, and (3) the duration of the RPA-CRISPR reaction. Initially, the molar ratio of Cas12a to crRNA was optimized, recognizing that the formation of a stable and catalytically active Cas12a-crRNA ribonucleoprotein (RNP) complex is a prerequisite for efficient cis-cleavage of target amplicons and collateral trans-cleavage of reporter probes in the biosensing cascade. An inappropriate molar ratio could compromise the structural stability of the RNP complex and diminish its cleavage efficiency. As shown in Figure 4A, the fluorescence intensities exhibited significant variation at the initial stage of the reaction. The system with a 2:3 molar ratio (purple curve) displayed the most substantial increase in fluorescence, suggesting the highest level of trans-cleavage activity. This finding was further validated by end-point fluorescence measurements at 120 cycles, where the 2:3 ratio produced the strongest signal (Figure 4B). This optimized ratio not only ensures maximal signal amplification for enhanced MH biosensing detection sensitivity but also provides a robust kinetic basis for further system optimization.

Subsequently, the volume of RPA amplicon added to the CRISPR reaction was evaluated. Given that the RPA product solution exhibits high ionic strength, it may influence Cas12a cleavage activity and impair AuNP-based chromogenic performance in the lateral flow assay. As depicted in Figure 4C, the fluorescence intensity increased progressively with the addition of 1 to 4 µL of the RPA product. However, a dramatic decline in fluorescence signal intensity was observed when the volume exceeded 4 µL, which is likely attributable to the excessive ionic strength disrupting the Cas12a-crRNA RNP complex structure and inhibiting its catalytic activity.

**Figure 5:**
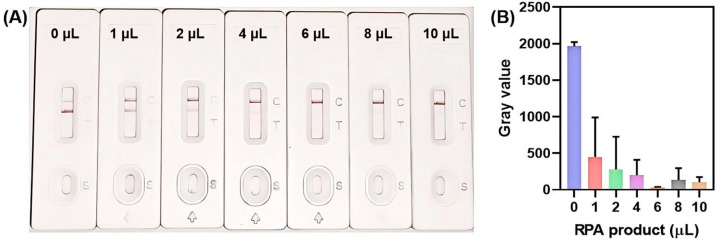
Optimization of RPA amplicon volume for lateral flow biosensor strips. (**A**) Visual detection results of lateral flow biosensors with different RPA amplicon volumes. (**B**) Gray values corresponding to the T zone signal of each amplicon volume. Error bars represent the standard deviation from three independent tests.

Furthermore, the final detection results were cross-validated with the lateral flow strips, which prompted a dedicated investigation into the impact of RPA amplicon volume on the chromogenic performance of the lateral flow biosensor. As depicted in Figure 5, both the color intensity of the T zone and the corresponding gray values demonstrated a characteristic biphasic response trend, decreasing initially and then increasing with the increase in RPA amplicon loading volume. Notably, when the loading volume exceeded 6 µL, an unexpected increase in the T zone’s color intensity was observed. This phenomenon can be attributed to the progressive elevation of ionic strength associated with increasing volumes of RPA amplicon solution. Given that AuNPs are highly sensitive to high ionic concentrations, such conditions can induce partial aggregation of AuNPs into larger colloidal clusters. These aggregated AuNP clusters are sterically trapped at the T zone, inhibiting their further migration and thereby leading to localized accumulation that enhances the visual chromogenic intensity.

Additionally, it was noted that the optimal RPA amplicon loading volume differed between the fluorescence-based method (4 µL) and the lateral flow strip-based visual detection method (6 µL). Since the assay ultimately relies on lateral flow strips for on-site, user-friendly visual readout, a loading volume of 6 µL was selected as the optimal parameter for all subsequent MH biosensing experiments.

An inadequate duration of Cas12a-mediated probe cleavage results in limited probe fragmentation, thereby undermining the ability to distinguish between positive and negative signals. Consequently, extending the reaction time of the RPA-CRISPR/Cas12a system is crucial to ensure complete bio-dig reporter probe cleavage and robust signal differentiation. Within this system, target recognition by crRNA triggers a cascade of enzymatic activities: Cas12a first performs cis-cleavage on the target nucleic acid, which is subsequently followed by trans-cleavage of the bio-dig probes. Since the degree of probe fragmentation serve as the primary basis for generating distinguishable positive/negative signals in the lateral flow biosensor, optimizing the duration of Cas12a cleavage is essential for reliable target identification.

**Figure 6:**
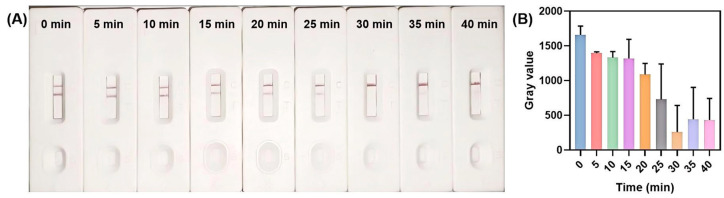
Optimization of reaction time for RPA-CRISPR/Cas12a system. (**A**) Visual detection results for various cleavage durations. (**B**) Quantitative gray value measurements of the T zone signal over different reaction times.

As shown in Figure 6, the gray value of the T zone decreased progressively with extended cleavage time, reaching its minimum at 30 min. This trend indicates that a 30 min cleavage duration is optimal for Cas12a-mediated reporter probe digestion, yielding the most reliable and distinguishable biosensing outcomes for MH detection. It is noteworthy that a slight increase in color intensity within the T zone occurs when the reaction time surpasses 30 min. We propose that extended reaction times result in the over-digestion of reporter probes into mono- or short-fragment DNA strands. At a pH of 9.0, SA possesses a net negative charge. In contrast, these truncated DNA fragments possess fewer negative charges than intact probes, resulting in reduced electrostatic repulsion between the fragments and the SA-modified T zone. This reduction may facilitate non-specific binding, causing these fragments to re-accumulate in the T zone. When AuNP-antibody complexes flows through the T zone, they may be captured by the digoxin-labeled mono- or short-fragment DNA, thereby causing an unintended increased color intensity in the T zone and blurring the boundary between positive and negative signals. Based on these findings, a reaction time of 30 min was determined to be optimal for the RPA-CRISPR/Cas12a system.

### 3.5. Specificity Validation of the MH Biosensing Assay

**Figure 7:**
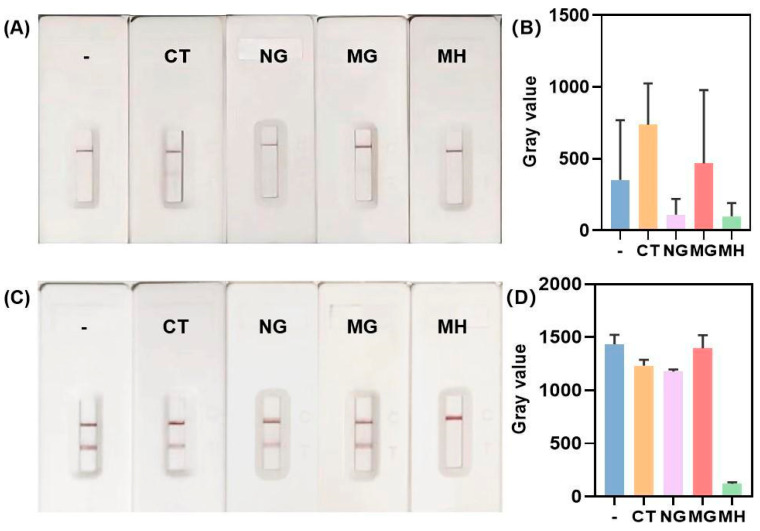
Specificity evaluation of the MH biosensing assay before and after aerosol contamination treatment. (**A**) Visual detection outcomes for different pathogens with aerosol contamination. (**B**) Quantitative detection outcomes for different urogenital pathogens under aerosol contamination conditions. (**C**) Visual detection results of each pathogen following the decontamination protocol. (**D**) Quantitative detection results for each pathogen post-treatment. Error bars represent standard deviations from three independent experimental replicates.

Commercially available certified liquid quality control materials for CT, NG, and MG were employed to evaluate the detection specificity of the developed MH biosensing assay. Establishing an aerosol-contamination-free workflow is crucial for ensuring the accuracy and reproducibility of nucleic acid-based biosensing assays, especially for MH detection where low-abundance target and amplicon carryover can cause false positives. The current integrated RPA-CRISPR/Cas12a-lateral flow assay is predominantly constrained by the necessity to open reaction tubes for transferring RPA amplicons into the CRISPR reaction system, a step that poses a significant risk of aerosol contamination from amplified nucleic acid fragments. In this study, non-negligible false-positive signals were observed in the MH specificity assay using CT, NG, and MG nucleic acids as controls (Figure 7A,B). As a result, the unoptimized procedure would have to be conducted in rigorously partitioned nucleic acid amplification laboratories with dedicated pre- and post-amplification zones. Such specialized facilities, however, are often unavailable in primary care hospitals and resource-limited remote areas.

Therefore, a series of targeted strategies for eliminating aerosol contamination were systematically evaluated. Preliminary findings revealed that although opening RPA reaction tubes within a biological safety cabinet (BSC) an reduce external contamination, the process itself introduces amplicon aerosols into the BSC interior, which can cross-contaminate subsequent experiments and compromise assay reliability. Consequently, it is imperative that the BSC undergoes a comprehensive decontamination procedure prior to its use in subsequent experiments. The detailed, optimized aerosol decontamination protocol is as follows: (1) 60 min of UV irradiation; (2) application of 1% sodium hypochlorite spray inside the BSC for 10 min; (3) application of a commercial nucleic acid decontamination solution spray for 10 min; (4) a second round of 1% sodium hypochlorite treatment for another 10 min; and (5) a final surface cleaning with deionized water. This optimized protocol was verified to be highly effective in eliminating aerosol contamination from RPA amplicons. As illustrated in Figure 7C,D, following implementation of the decontamination protocol, a distinct red band was observed at the T zone for CT, NG, and MG nucleic acid extracts, whereas the red band at the T zone was scarcely visible to the naked eye for MH nucleic acid extracts. These results demonstrate the high specificity of the optimized biosensing assay, with no cross-reactivity to other three clinically relevant urogenital pathogens.

### 3.6. Sensitivity Validation of the MH Biosensing Assay

In this study, recombinant plasmid standards containing MH 16SrRNA gene were employed as quantification standards to evaluate the detection sensitivity of the developed MH biosensing assay. Recombinant plasmids were selected as the sensitivity testing templates due to their superior structural stability compared to commercial liquid quality control materials, which ensures batch-to-batch consistency of target nucleic acid concentration and eliminates matrix interference from clinical or microbial lysates, rendering them far more suitable for precise sensitivity characterization of nucleic acid-based biosensors.

**Figure 8:**
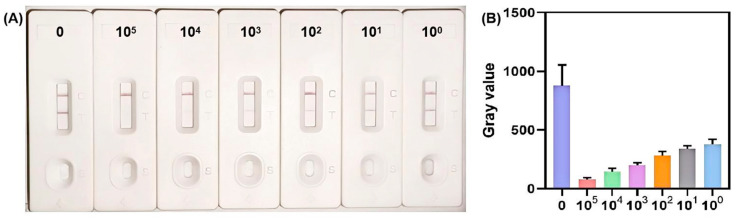
Sensitivity evaluation of the MH biosensing assay using recombinant plasmid standards. (**A**) Visual detection results for serial dilutions of MH 16S rRNA gene recombinant plasmid (copies/μL). (**B**) Quantitative analysis of T zone signals for different plasmid concentrations (copies/μL). Error bars represent standard deviations from three independent experimental replicates. The volume for each concentration of recombinant plasmid standards was 2 μL/reaction.

A 10-fold serial dilution series of the recombinant plasmid was prepared, ranging from 10^5^ to 1 copies/μL. As shown in Figure 8A, a dose-dependent increase in color intensity of the T zone was observed with decreasing plasmid concentrations. This trend correlates directly with the reduction in Cas12a-mediated cleavage of the bio-dig reporter probe. Upon visual inspection, the colorimetric result for the 2 copies/reaction recombinant plasmid was distinctly distinguishable from the negative control (no plasmid template). Statistical analysis further confirmed that the gray values of samples at 2 copies/reaction exhibited a significant difference from those of negative samples (Figure 8B). Therefore, the optimized biosensing method achieves a limit of detection (LOD) of 2 copies/reaction recombinant plasmid containing target gene for both visual readout and quantitative signal analysis.

Meanwhile, we notice that when the concentration of MH in a sample is very low, visual interpretation of the lateral flow strip alone can be challenging. In such low-target scenarios, quantitative methods like qPCR remain the recommended choice for definitive detection. Consequently, our visual RPA-CRISPR/Cas12a assay is particularly well-suited for the rapid screening of samples with moderate to high bacterial loads.

### 3.7. Clinical Sample Validation of the MH Biosensing Assay

As previously described, this study initially validated the MH biosensing assay using certified liquid quality control materials and recombinant plasmids, which are characterized by high nucleic acid purity and negligible matrix interference. However, clinical samples exhibit far greater complexity, as they contain abundant human genomic DNA, and other biological impurities that may cause non-specific binding or enzyme inhibition, thereby compromising detection accuracy and reproducibility. Consequently, clinical validation is indispensable for verifying the applicability of the developed biosensing method to real-world diagnostic scenarios.

**Figure 9:**
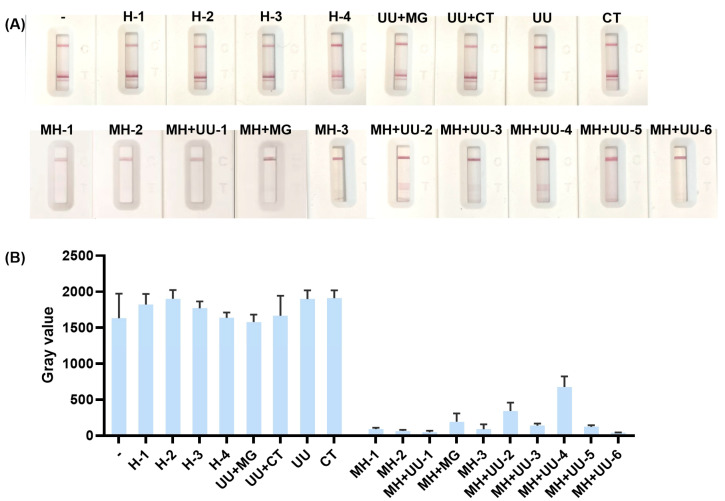
Clinical validation results of the MH biosensing assay using 18 clinical specimens. (**A**) Visual detection readouts of clinical samples. (**B**) Quantitative grayscale value analysis of T zone signals for clinical samples.

We tested 18 clinical specimens, including four healthy (H) specimens, one UU and MG coinfected specimen, one UU and CT coinfected specimen, one UU-monoinfected clinical specimen, one CT-monoinfected clinical specimen, three MH-monoinfected clinical specimens, six MH and UU coinfected specimen, and one MH and MG coinfected specimen. As shown in Figure 9, the color intensity of the T zone for healthy specimens, UU or CT-monoinfected specimens, and clinical specimens co-infected with UU and MG or UU and CT showed no significant difference compared to the negative control. In contrast, a clear reduction in T zone color was observed for both MH-monoinfected specimens and specimens co-infected with MH and UU or MH and MG. This signal trend is consistent with the assay’s core mechanism: the presence of MH target nucleic acid activates Cas12a’s collateral trans-cleavage activity, which degrades the bio-dig reporter probe and reduces the amount of intact probe available for streptavidin-mediated capture at the T zone. These results confirm the excellent specificity of the developed biosensing system for MH detection and its effective recognition of target pathogens in clinical specimens, demonstrating its strong potential for clinical application.

## 4. Discussion

This study presents the development of a lateral flow strip-based nucleic acid biosensor that integrates RPA and CRISPR/Cas12a technologies for the rapid detection of MH. Significantly, this work presents the first MH-specific CRISPR-LFA method that incorporates a documented contamination-control protocol. The integrated biosensing platform enables visual detection of MH at a LOD as low as 2 copies/reaction of recombinant plasmid containing the MH 16SrRNA gene with a total assay time of 60 min. And the detection limit and assay time of this lateral flow biosensor-based CRISPR/Cas12a assay for MH detection are comparable to those of recently reported methods (Table S2) [17,23,24,25,26,27,28,29,30,31]. While the detection results from 18 clinical specimens demonstrated excellent specificity for MH, future studies with a larger cohort are warranted to comprehensively evaluate its clinical diagnostic performance.

This robust and visible detection system is uniquely suited for scenarios requiring rapid or simplified testing, where access to sophisticated molecular diagnostic equipment is often restricted. Notably, the incorporation of a dedicated aerosol contamination control strategy significantly enhances the feasibility of deploying this detection method in simplified settings, while upholding stringent quality control standards for nucleic acid amplification assays. The multi-step decontamination protocol effectively eliminates aerosol contamination from RPA amplicons. Unlike fluorescence-based detection methods, the RPA/CRISPR-based lateral flow assay requires precise optimization of reaction time to prevent false-negative results, as over-prolonged incubation can induce non-specific probe fragmentation and blunted signal differentiation between positive and negative samples.

Although a variety of one-tube (closed) RPA/CRISPR detection methods have been established for nucleic acid diagnostics [30,32,33,34], the necessity to open reaction tubes persists when these assays are coupled with lateral flow strip readout for visual POC detection. Future research endeavors will focus on developing a fully integrated nucleic acid detection system that merges one pot RPA/CRISPR reactions with a closed lateral flow biosensor format. This next-generation platform will eliminate the need for open-tube sample transfer, thereby fundamentally mitigating the risk of aerosol contamination during operational processes.

## Data Availability

Data are contained within the article or Supplementary Materials.

## Supplementary Materials

The following supporting information can be downloaded at https://www.mdpi.com/article/10.3390/bios15120821/s1, Figure S1: Characterization of gold nanoparticles; Figure S2: Electrophoresis of RPA amplicons with three primer pairs; Table S1: Sequences of three RPA primer sets, crRNAs, and reporter probes used in this manuscript.
