# Supplementary material for: Rapid Visual Detection of Mycoplasma Hominis Using an RPA-CRISPR/Cas12a Assay
Source: Biosensors (Basel). 2025 Dec 18;15(12):821. doi: 10.3390/bios15120821 (PMC12730632; doi:10.3390/bios15120821)
Supplement: Supplementary file 1 [file biosensors-15-00821-s001.zip › biosensors-3991453-supplementary.pdf]

biosensors

MDPI

Supplementary Materials

Rapid Visual Detection of Mycoplasma hominis Using an RPA-CRISPR/Cas12a Assay

Jie Chen [1,†], Shutao Liu [2,†], Sunyi Chen [3,†], Jingwen Mai [4], Maiwula Abudukadi [4], Yao Chen [4], Jie Lu [2], Guanglei Li [5,*] and Chenchen Ge [1,*]

1 College of Health Science and Environmental Engineering, Shenzhen Technology University, 3002 Lantian Road, Pingshan District, Shenzhen 518118, China; 202200501096@stumail.sztu.edu.cn(J.C.)
2 Department of Radiology and Nuclear Medicine, Xuanwu Hospital, Capital Medical University, Beijing 100053, China; lst41@sina.com (S.L.); imaginglu@hotmail.com (J.L.)
3 Department of Dermatology, Huashan Hospital, Fudan University, Shanghai 200040, China; csyysc28@163.com (S.Y.C.)
4 KingMed School of Laboratory Medicine, Guangzhou Medical University, Guangzhou 511436, China; 2023112115@stu.gzhmu.edu.cn (J.M.); 2021141087@stu.gzhmu.edu.cn (M.A.); 2023112113@stu.gzhmu.edu.cn (Y.C.)
5 College of Pharmacy, Shenzhen Technology University, 3002 Lantian Road, Pingshan District, Shenzhen 518118, China
* Correspondence: liguanglei@sztu.edu.cn (G.L.); gechenchen@sztu.edu.cn (C.G.)
† Jie Chen, Shutao Liu, and Sunyi Chen contributed equally to this work.

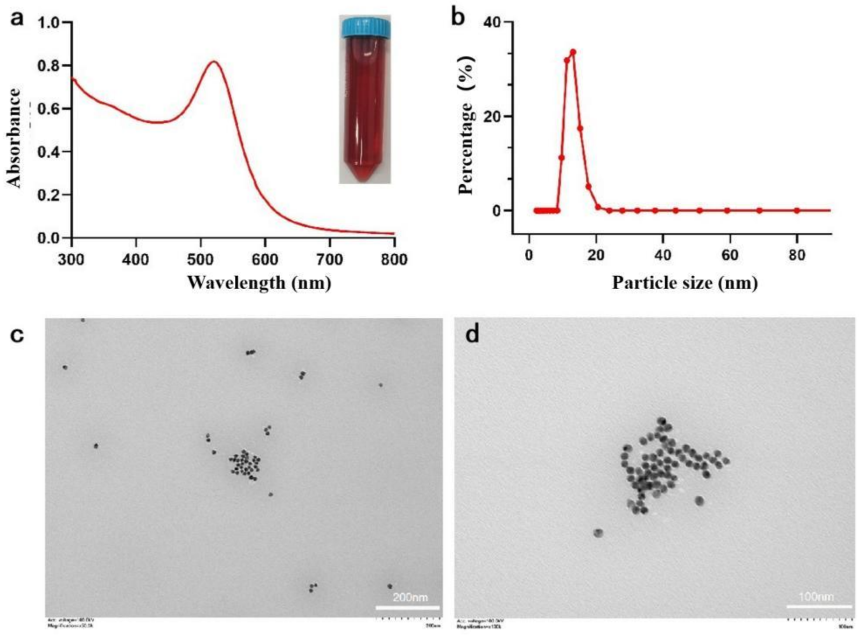

Figure S1. Characterization of gold nanoparticles. (**a**) UV-vis absorption spectra of colloidal gold nanoparticles from three independent measurements; (**b**) Size distribution profile; Transmission electron microscopy (TEM) images with 200nm(**c**) and 100nm(**d**) scale bars.

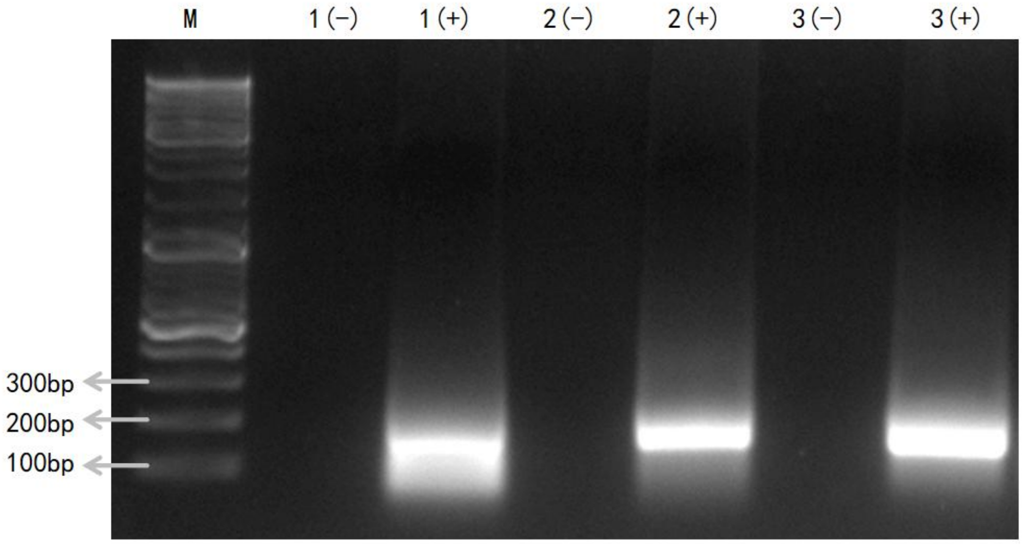

Figure 2. Electrophoresis of RPA amplicons with three primer pairs. Lane M shows the DNA marker; Lanes 1 (-) and 1 (+) are the negative control and positive sample for primer set 1; Lanes 2 (-) and 2 (+) for primer set 2; Lanes 3 (-) and 3 (+) for primer set 3.

Table S1. Sequences of three RPA primer sets, crRNAs, and reporter probes used in this manuscript.

Names	sequence（5’-3’）	Product size
RPA Forward Primer1	GATAGAGGTAAGCGGAATTCCATGTGAAGC	174bp
RPA Reverse Primer1	CCGACTAATGATCATCGTTTACGGCGTGGAC	
crRNA1	UAAUUUCUACUAAGUGUAGAUACCGCUUCACAUGGAAUUCC	
RPA Forward Primer2	ATGACGTCAAATCATCATGCCTCTTACGAG	172bp
RPA Reverse Primer2	GATCTGCGATTACTAGCGATTCCGACTTCAT	
crRNA2	UAAUUUCUACUAAGUGUAGAUUACCGACCAUUGUAGCACGU	
RPA Forward Primer3	CATGTGAAGCGGTGAAATGCGTAGATATATGG	154bp
RPA Reverse Primer3	CCGACTAATGATCATCGTTTACGGCGTGGAC	
crRNA3	UAAUUUCUACUAAGUGUAGAUCGGCGUGGACUACCAGGGU	
FQ reporter probe	FAM-TTTATTTATTTT-BHQ1	
Bio-dig reporter probe	Digoxin-TTTATTTATTTT-Biotin	

Table S2. Recent articles (published between 2020 and 2025) on pathogen detection with lateral flow biosensor-based CRISPR/Cas systems.

Method	Target	Assay time	Sensitivity	Reaction System	Reference
RT-LAMP-CRISPR/Cas12-LFA	SARS-CoV-2	40 min	10 copies/μL	Two step	[1]
RPA-CRISPR-Cas12a/13a	HPV 16, 18	40 min	10 copies/μL	Two step	[2]
RPA-CRISPR/Cas12a	Neisseria gonorrhoeae	60 min	5 pg/μl	Two step	[3]
Multiplex Integrated RPA-CRISPR/Cas12a detection Assay (MIRCA)	Neisseria gonorrhoeae & resistance genes	40 min	10-20 copies/reaction	Two step	[4]
HPV-multiple RPA-CRISPR/Cas12a	Six HPV types	45 min	1 copy/μl	Two step	[5]
Cas12a cis-cleavage mediated lateral flow assay (cc-LFA)	Nine HPV types & multiple respiratory pathogen	N/A	20 copies/reaction	Two step	[6]
RPA-CRISPR-LbaCas12a-LFD	Prymnesium parvum	N/A	High	Two step	[7]
RPA-CRISPR/Cas12a (fluorescence/LFS)	Trichomonas vaginalis	60 min	1 copy/μl	One pot	[8]
RPA-CRISPR/Cas12a-LFA	Monkeypox	40 min	10 aM	One pot	[9]
RPA-CRISPR Cas12a/Cas13a	MRSA	30 min	5 copies/μl	One pot	[10]

References

1. Broughton, J.P.; Deng, X.; Yu, G.; Fasching, C.L.; Servellita, V.; Singh, J.; Miao, X.; Streithorst, J.A.; Granados, A.; Sotomayor-Gonzalez, A.; et al. CRISPR–Cas12-based detection of SARS-CoV-2. Nat. Biotechnol. **2020**, *38*, 870–874, https://doi.org/10.1038/s41587-020-0513-4.
2. Zhang, K.; Li, Q.; Wang, K.; Zhang, Q.; Ma, C.; Yang, G.; Xie, Y.; Mauk, M.G.; Fu, S.; Chen, L. RPA-CRISPR-Cas-Mediated Dual Lateral Flow Assay for the Point-of-Care Testing of HPV16 and HPV18. Bioconjugate Chem. **2024**, *35*, 1797–1804, https://doi.org/10.1021/acs.bioconjchem.4c00375.
3. Tu, Q.; Cao, X.; Ling, C.; Xiang, L.; Yang, P.; Huang, S. Point-of-care detection of Neisseria gonorrhoeae based on RPA-CRISPR/Cas12a. AMB Express **2023**, *13*, 50. https://doi.org/10.1186/ s13568-023-01554-7.
4. Zhao, Z.; Li, Y.; Xiu, L.; Wang, F.; Peng, J. Development of a CRISPR/Cas-based detection platform for tracking decreased susceptibility to cephalosporins in Neisseria gonorrhoeae. Anal. Chem. **2025**, 97, 19445–19455. https://doi.org/10.1021/acs.analchem.5c01400.
5. Liu, Y.; Chao, Z.; Ding, W.; Fang, T.; Gu, X.; Xue, M.; Wang, W.; Han, R.; Sun, W. A multiplex RPA-CRISPR/Cas12a-based POCT technique and its application in human papillomavirus (HPV) typing assay. Cell. Mol. Biol. Lett. **2024**, *29*, 34. https://doi.org/10.1186/s11658-024-00548-y.
6. Lin, M.; Qiu, Z.; Hao, M.; Qi, W.; Zhang, T.; Shen, Y.; Xiao, H.; Liang, C.; Xie, L.; Jiang, Y.; et al. Cas12a Cis-cleavage mediated lateral flow assay enables multiplex and ultra-specific nucleic acid detection. Nat. Commun. **2025**, *16*, 5597. https://doi.org/10.1038/s41467-025-60917-9.
7. Huang, H.-L.; Luo, N.-J.; Chen, W.-Z.; Wang, X.-W.; Zhou, C.-X.; Jiang, H.-B. A highly specific and ultrasensitive approach to detect Prymnesium parvum based on RPA-CRISPR-LbaCas12a-LFD system. Anal. Chim. Acta **2024**, *1315*, 342797, https://doi.org/10.1016/j.aca.2024.342797.
8. Li, S.; Wang, X.; Yu, Y.; Cao, S.; Liu, J.; Zhao, P.; Li, J.; Zhang, X.; Li, X.; Zhang, N.; et al. Establishment and application of a CRISPR-Cas12a-based RPA-LFS and fluorescence for the detection of Trichomonas vaginalis. Parasites Vectors **2022**, *15*, 350, https://doi.org/10.1186/s13071-022-05475-5.
9. Liu, S.; Yang, Y.; Li, X.; Choi, J.-W.; Guo, J.; Luo, H.; Li, C. Development of a single-tube RPA/CRISPR-cas12a detection platform for monkeypox virus. Biosens. Bioelectron. **2025,** *278*, 117221, https://doi.org/10.1016/j.bios.2025.117221.
10. Liu, Y.; Liu, H.; Yu, G.; Sun, W.; Aizaz, M.; Yang, G.; Chen, L. One-tube RPA-CRISPR Cas12a/Cas13a rapid detection of methicillin-resistant Staphylococcus aureus. Anal. Chim. Acta **2023,** *1278*, 341757, https://doi.org/10.1016/j.aca.2023.341757.
